# Effects of One-Year Tofacitinib Therapy on Lipids and Adipokines in Association with Vascular Pathophysiology in Rheumatoid Arthritis

**DOI:** 10.3390/biom12101483

**Published:** 2022-10-14

**Authors:** Monika Czókolyová, Attila Hamar, Anita Pusztai, Gábor Tajti, Edit Végh, Zsófia Pethő, Nóra Bodnár, Ágnes Horváth, Boglárka Soós, Szilvia Szamosi, Anita Szentpéteri, Ildikó Seres, Mariann Harangi, György Paragh, György Kerekes, Levente Bodoki, Andrea Domján, Katalin Hodosi, Tamás Seres, György Panyi, Zoltán Szekanecz, Gabriella Szűcs

**Affiliations:** 1 Department of Rheumatology, University of Debrecen, 4032 Debrecen, Hungary; 2 Department of Biophysics and Cell Biology, University of Debrecen, 4032 Debrecen, Hungary; 3 Division of Metabolic Diseases, University of Debrecen, 4032 Debrecen, Hungary; 4 Intensive Care Unit, Department of Medicine, Faculty of Medicine, University of Debrecen, 4032 Debrecen, Hungary; 5 Department of Anesthesiology, University of Colorado Anschutz Medical Campus, Aurora, CO 80045, USA

**Keywords:** rheumatoid arthritis, tofacitinib, JAK inhibitors, lipids, adipokines

## Abstract

Background: Cardiovascular (CV) morbidity, mortality and metabolic syndrome are associated with rheumatoid arthritis (RA). A recent trial has suggested increased risk of major CV events (MACE) upon the Janus kinase (JAK) inhibitor tofacitinib compared with anti-tumor necrosis factor α (TNF-α) therapy. In our study, we evaluated lipids and other metabolic markers in relation to vascular function and clinical markers in RA patients undergoing one-year tofacitinib therapy. Patients and methods: Thirty RA patients treated with either 5 mg or 10 mg bid tofacitinib were included in a 12-month follow-up study. Various lipids, paraoxonase (PON1), myeloperoxidase (MPO), thrombospondin-1 (TSP-1) and adipokine levels, such as adiponectin, leptin, resistin, adipsin and chemerin were determined. In order to assess flow-mediated vasodilation (FMD), common carotid intima-media thickness (IMT) and arterial pulse-wave velocity (PWV) ultrasonography were performed. Assessments were carried out at baseline, and 6 and 12 months after initiating treatment. Results: One-year tofacitinib therapy significantly increased TC, HDL, LDL, APOA, APOB, leptin, adipsin and TSP-1, while significantly decreasing Lp(a), chemerin, PON1 and MPO levels. TG, lipid indices (TC/HDL and LDL/HDL), adiponectin and resistin showed no significant changes. Numerous associations were found between lipids, adipokines, clinical markers and IMT, FMD and PWV (*p* < 0.05). Regression analysis suggested, among others, association of BMI with CRP and PWV (*p* < 0.05). Adipokines variably correlated with age, BMI, CRP, CCP, FMD, IMT and PWV, while MPO, PON1 and TSP-1 variably correlated with age, disease duration, BMI, RF and PWV (*p* < 0.05). Conclusions: JAK inhibition by tofacitinib exerts balanced effects on lipids and other metabolic markers in RA. Various correlations may exist between metabolic, clinical parameters and vascular pathophysiology during tofacitinib treatment. Complex assessment of lipids, metabolic factors together with clinical parameters and vascular pathophysiology may be utilized in clinical practice to determine and monitor the CV status of patients in relation with clinical response to JAK inhibition.

## 1. Introduction

The Janus Kinase (JAK) family of tyrosine kinases are transducers of cytokine signaling [1,2]. JAK inhibitors have demonstrated efficacy and safety in patients with rheumatoid arthritis (RA) [1,3,4,5]. As of today, four orally administered JAK inhibitors, tofacitinib, baricitinib, upadacitinib and filgotinib have been approved for the treatment of RA [6].

RA has been associated with inflammatory atherosclerosis and increased cardiovascular (CV) morbidity and mortality, as well as dyslipidemia [7,8,9,10,11,12,13]. In RA, a “lipid paradox” has been described with decreased TC and LDL levels, which showed association with inflammation and higher CV risk. Thus, immunosuppressive therapy that dampens inflammation and CRP may, in turn, increase lipid levels [14]. Tofacitinib increases LDL and HDL levels [5,15,16].

Adipokines have been associated with the pathogenesis of RA and its comorbidities [8,17]. There are a number of adipokines; here, we only mention those included in this study. Among adipokines, adiponectin has anti-inflammatory and anti-atherogenic properties [17,18]. However, in some diseases, such as RA, adiponectin seems to have pro-inflammatory effects as it stimulates chronic inflammation by various actions [17,18,19]. In this regard, in a former study in patients with RA undergoing infliximab therapy due to severe disease, high-grade inflammation was independently and negatively correlated with circulating adiponectin concentrations. In contrast, low plasma adiponectin levels cluster with metabolic syndrome features that contribute to atherogenesis in RA. With respect to this, plasma adiponectin concentrations negatively correlated with triglycerides/HDL cholesterol ratios, total cholesterol/HDL cholesterol ratios and high fasting plasma glucose levels, independently of CRP levels and the BMI [20]. Leptin is pro-inflammatory and pro-atherogenic [8,17], chemerin is a chemoattractant with a pro-inflammatory and pro-atherogenic nature [21,22], while resistin is also of a pro-inflammatory nature [17,23]. Adipsin, identified as complement factor D, is a serine protease [24]. Its pro- or anti-inflammatory nature has not yet been fully established; further studies are needed in this regard [25]. Increased levels of adiponectin, leptin, resistin and chemerin have been reported in RA patients by various research groups (reviewed in [8,11,17]. Baseline serum adipokine levels have been associated with radiographic progression in RA [8,26]. Targeted therapies may influence adipokine levels in RA; however, the results have been somewhat controversial [8,17,23,27].

Myeloperoxidase (MPO) is a heme-containing peroxidase most abundantly found in neutrophils. MPO is involved in neutrophil oxidative burst and has been associated with atherosclerosis, the development of unstable plaques and CV disease [28,29]. There are increased plasma MPO levels in RA [28,30]. MPO is also involved in RA-related oxidative stress [28]. In the extracellular matrix (ECM), MPO works as a NO-oxidase, leading to impaired endothelial relaxation [29]. We have found that anti-TNF treatment might decrease MPO levels in RA [27].

Thrombospondin-1 (TSP-1) is a glycoprotein involved in angiogenesis, inflammation, atherogenesis and RA pathogenesis, which is suggested to have pro-inflammatory and pro-atherogenic properties [31,32]. Elevated TSP-1 levels have been reported in RA [31,33]. TSP-1 exerts angiostatic properties [32].

Paraoxonase 1 (PON1) is an esterase enzyme of antioxidant, anti-atherogenic and anti-inflammatory properties [34,35]. Apart from its PON activity, PON1 also exerts arylesterase (ARE) activity [35,36]. Impaired PON1 PON and ARE activity has been found in inflammatory diseases associated with accelerated atherosclerosis, such as RA [37,38]. In RA, there was an inverse correlation between disease activity and PON1 [39]. We have found a correlation between PON activity and serum TNF-α levels in RA suggesting that PON1 production may be a result of a feedback response to cytokine release [10]. Biologics may alter PON and ARE activity in RA [27,40].

Surrogate markers of CV disease have been introduced to determine vascular pathophysiology in RA [9]. Among ultrasound-based imaging techniques, common carotid intima-media thickness (IMT), brachial artery flow-mediated vasodilation (FMD) and arterial pulse-wave velocity (PWV) are suitable to detect overt atherosclerosis, endothelial dysfunction and vascular stiffness, respectively [9,10]. RA has been associated with increased IMT and PWV, as well as impaired FMD [9,10,13,41,42,43]. Targeted therapies might, at least transiently, improve FMD, IMT and PWV (reviewed in [44]).

There have been relatively few studies on the effects of tofacitinib on metabolic biomarkers including lipids and adipokines, metabolic syndrome (MS) and CV disease. JAK inhibitors might increase lipid levels without changing the atherogenic index primarily due to the lipid paradox [7,45,46]. Tofacitinib generally did not increase CV risk in the clinical program [5,47,48]. Tofacitinib was associated with a low incidence of cardiovascular events in a large Phase 3 program, including long-term extension studies [49]. However, as found in the recent ORAL Surveillance study, tofacitinib might increase the risk of MACE in RA patients in comparison with TNF-α inhibitors [50]. Yet, there was no difference between tofacitinib and anti-TNF therapy in lipid elevations, and the increased risk of MACE was not associated with lipid changes [50]. With respect to adipokines, there have been no publications on the possible effects of tofacitinib on leptin, adiponectin, chemerin, adipsin or resistin. With respect to vascular pathology, we have recently reported in the very same cohort that tofacitinib dampened aortic wall inflammation by PET/CT [51]. There has been only one additional study assessing IMT in tofacitinib-treated patients [52]. Recently, in the same patient cohort assessed in the present study, one-year tofacitinib treatment prevented the worsening of FMD and PWV, while IMT still progressed despite treatment [53]. These findings are reminiscent of those previously reported using anti-TNF agents. In this sense, a rapid improvement in endothelial function was observed after a single infusion of the anti-TNF agent infliximab [54]. However, progression of subclinical atherosclerosis, manifested by an increase in the carotid IMT, was found despite treatment with this anti-TNF agent [55]. Otherwise, no data have become available on the effects of JAK inhibition on vascular pathophysiology.

To our best knowledge, no other studies have been conducted on the effects of JAK inhibitors, namely those of tofacitinib, on metabolic markers including lipids, adipokines, MPO, PON1 or TSP-1 in association with clinical parameters and vascular pathophysiology as determined by FMD, PWV and IMT in RA. Therefore, we conducted a one-year, prospective study in order to assess the effects of tofacitinib on inflammation, vascular pathophysiology and metabolic markers. We previously assessed the effects of tofacitinib on arterial inflammation, FMD, IMT and PWV, as well as arginine and methionine metabolites in the very same cohort [51,53]. By performing this study, we wished to determine the surrogate markers of tofacitinib effects on CV pathology.

## 2. Patients and Methods

### 2.1. Patients and Study Design

Thirty patients with active RA were recruited for this tofacitinib interventional study. Patient characteristics are presented in Table 1. The study population included 27 women and 3 men with a mean age of 52.8 ± 10.0 (range: 27–69) years. Mean disease duration was 7.7 ± 5.0 (range: 1–21) years. Mean baseline DAS28 was 5.05 ± 0.77 (4.80 ± 0.69 and 5.29 ± 0.79 in the 5 mg bid and 10 mg bid treatment arm, respectively). There was a slight difference in DAS28 between the 5 mg bid vs 10 mg bid arms; however, this might not have clinical relevance. Yet, according to the “lipid paradox”, systemic inflammation and disease activity may inversely correlate with lipid levels [14]. Mean BMI was 29.93± 6.90. Eighty percent of patients (*n* = 24) were rheumatoid factor positive and 80% were ACPA positive (*n* = 24) (Table 1).

Patients who met the inclusion criteria of the definitive diagnosis of RA according to the 2010 European League Against Rheumatism (EULAR)/American College of Rheumatology (ACR) classification criteria for RA [56]; moderate-high disease activity (DAS28 > 3.2) at baseline and clinical indication of targeted therapy and had neither of the following exclusion criteria were included. The exclusion criteria included inflammatory diseases other than RA, acute/recent infection, standard contraindications to JAK inhibition, uncontrolled CV disease or hypertension, and chronic renal or liver failure and malignancy within the last 10 years. Patients were either naive to any targeted therapies (*n* = 16) or switched to tofacitinib after stopping a biologic and an appropriate washout period had passed (*n* = 14).

Half of the patients (*n* = 15) were randomly assigned to a 5 mg tofacitinib twice daily (bid) treatment arm; the other half (*n* = 15) were assigned to a 10 mg tofacitinib twice daily (bid) treatment arm. Supplemental therapy of either methotrexate (MTX) (*n* = 16), sulfasalazine (*n* = 1), leflunomide (*n* = 4), MTX + sulfasalazine (*n* = 1) or leflunomide + sulfasalazine (*n* = 1) were given to patients (Table 1). DMARDs were taken in stable doses at least one year prior to the present study. No dose changes of these DMARDs were allowed throughout the course of the study. Although most patients may have received corticosteroids prior to the study, none of the patients had been on corticosteroids for at least 3 months prior to or during the study. Regarding lipid-lowering therapies, only one patient received such therapy; no dose changes occurred during the study.

Clinical assessments were performed at baseline, and after 6 and 12 months of therapy. During the study, a total of 4 patients, 2-2 on each treatment arm, dropped out after 6 months of treatment but before the end of the study. In 2 cases, the reason was inefficacy; in one case, significantly elevated transaminases were detected; and in the last case, the patient moved abroad. Altogether 26 patients (13-13 patients on each arm) completed the study and were thus eligible for further data analysis.

The study was approved by the Hungarian Scientific Research Council Ethical Committee (approval No. 56953-0/2015-EKL). Written informed consent was obtained from each patient and assessments were carried out according to the Declaration of Helsinki and its amendments.

### 2.2. Clinical Assessment

First, a detailed medical history was taken. We inquired about the history of CVD, as well as current smoking, experience of chest pain resembling angina pectoris, hypertension and diabetes mellitus during the last 2 years prior to the start of this study by a questionnaire. Altogether, 6 patients (3-3 on each arm) had a positive CV history. A total of 15 patients had hypertension (5-10), 2 had diabetes mellitus (1-1) and 7 patients (4-3) were current smokers at the time of inclusion. Disease activity of RA was calculated as DAS28-CRP (3 variables). Body mass index (BMI) was calculated based on the patients’ height and weight. Obesity was defined as BMI > 30 kg/m^2^; a total of 10 patients were found to exceed this limit (Table 1). Further clinical assessments including physical examination were performed at baseline, and after 6 and 12 months of tofacitinib therapy.

### 2.3. Laboratory Measurements

Venous blood samples were taken after an overnight fast and sera were immediately prepared. Lipid analyses including TC, LDL-C, HDL-C, TG, lipoprotein(a) [Lp(a)], APOA and APOB were performed from fresh sera with a Cobas c501 autoanalyzer (Roche Ltd., Mannheim, Germany). Among adipokines, serum chemerin concentrations (ng/mL) were determined by commercially available ELISA kits (Human Chemerin ELISA Kit, Reagent Genie) with CV < 8% intra-, and CV < 10% inter-assay variabilities. Adiponectin, leptin, adipsin and resistin concentrations (pg/mL) were determined by flow cytometry by a bead-based multiplex assay using sera (Human Metabolic Panel 1, 4plex, LEGEND plex, BioLegend) and analyzed by LEGENDplex software (version 8.0). The leptin/adiponectin ratio as well as different lipid ratios (TC/HDL, LDL/HDL, APOA/APOB) were calculated.

Serum PON1 (ng/mL) concentrations were determined by commercially available ELISA kits (Human PON1/Paraoxonase 1 ELISA Kit, Reagent Genie). Serum MPO (ng/mL) concentrations were determined by commercially available ELISA kits (Human Myeloperoxidase ELISA Kit, Reagent Genie). Serum TSP-1 (micrograms per milliliter, μg/mL) concentrations were determined by commercially available ELISA kits (Human Thrombospondin-1 ELISA Kit, Reagent Genie). The previous ELISA kits had CV < 8% intra-, and CV < 10% inter-assay variabilities.

The erythrocyte sedimentation rate (ESR) (millimeter per hour, mm/h) was determined. Serum high sensitivity C reactive protein (hsCRP; normal: ≤5 mg/l) and IgM rheumatoid factor (RF; normal: ≤50 IU/mL) were measured by quantitative nephelometry (Cobas Mira Plus, Roche Diagnostics, Basel, Switzerland), using CRP and RF reagents (both Dialab Ltd., Budapest, Hungary). ACPA (CCP) autoantibodies were detected in serum samples using a second generation Immunoscan-RA CCP2 ELISA test (Euro Diagnostica, Malmö, Sweden; normal: ≤25 IU/mL). The assays were performed according to the manufacturer’s instructions.

### 2.4. Assessment of Vascular Physiology by Ultrasound

The brachial artery FMD, common carotid IMT and aortic PWV assessments carried out in the very same cohort were performed and previously published [10,27,41]. Two investigators (GK, EV) performed these assessments. Data on the tofacitinib effects on these parameters in this very same cohort have been previously presented [53]. In this study, FMD, IMT and PWV data were only used in the correlation analysis.

### 2.5. Statistical Analysis

Statistical analysis was performed using SPSS version 22.0 (IBM, Armonk, NY, USA) software. Data were expressed as the mean ± SD for continuous variables and percentages for categorical variables. The distribution of continuous variables was evaluated by the Kolmogorov-Smirnov test. Continuous variables were evaluated by the paired two-tailed t-test and Wilcoxon test. Nominal variables were compared between groups using the chi-squared or Fisher’s exact test, as appropriate. Correlations were determined by Pearson’s analysis. Univariate and multivariate regression analysis using the stepwise method were applied to determine independent metabolic determinants of FMD, IMT and PWV (dependent variables), as well as independent determinants of the studied metabolic parameters (lipids, adipokines, MPO, PON1 and TSP-1) (dependent variables). The β standardized linear coefficients showing linear correlations between two parameters were determined. The B (+95% CI) regression coefficient indicated independent associations between dependent and independent variables during changes. The general linear model (GLM) repeated measures analysis of variance (RM-ANOVA) was performed in order to determine the additional effects of multiple parameters including therapy on 0-6-12-month changes in metabolic parameters. In this analysis, partial η^2^ is given as an indicator of effect size, with values of 0.01 suggesting small, 0.06 medium and 0.14 large effects. The power was estimated using the G*-Power 3 software. *p* values < 0.05 were considered significant.

The reliability of the vascular ultrasound measurements was previously tested by inter-item correlation and intraclass correlation (ICC) before [10,41]. With respect to the FMD, IMT and PWV tests, ICC = 0.470; F-test value: 1.887; *p* = 0.001. The power was estimated using the G*- Power software [57]. *p* values < 0.05 were considered significant.

We also estimated the sample size needed. For example, with respect to the different adipokines, in the case of 6-month changes, a sample size of 30 resulted in significant changes at the 57–98% power. So, we think the sample size was enough to draw conclusions.

## 3. Results

### 3.1. Treatment Responses and Vascular Pathophysiology

The clinical efficacy of the same study has been reported before. In brief, treatment with tofacitinib, either 5 or 10 mg bid, significantly decreased CRP, DAS28 and improved HAQ after 6 and 12 months [51,53,58]. The effects of tofacitinib on vascular pathophysiology has also been presented. In general, FMD and PWV did not change, while IMT increased overtime [53]. Here we only used these data in order to correlate the measured lipids, adipokines and other metabolic parameters with them. Thus, none of the data to be presented below have been published.

### 3.2. Effects of Tofacitinib Therapy on Circulating Metabolic Biomarkers

**Figure 1:**
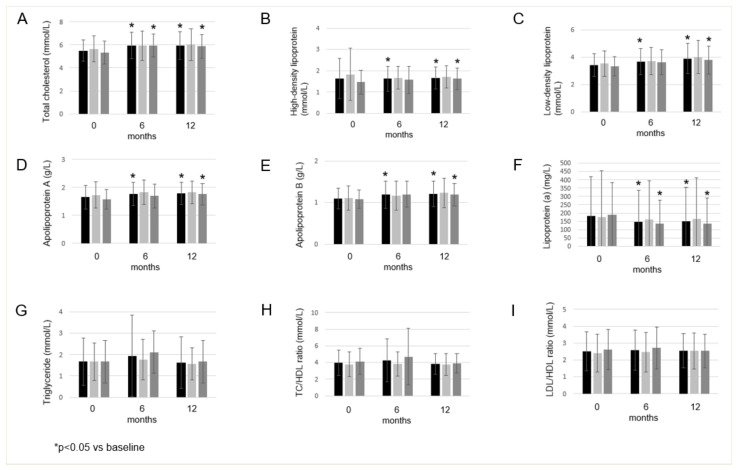
Effects of one-year tofacitinib therapy on total cholesterol (**A**), high-density lipoprotein (**B**), low-density lipoprotein (**C**), apolipoprotein A (**D**), apolipoprotein B (**E**), lipoprotein(a) (**F**) and triglyceride (**G**) levels, as well as TC/HDL (**H**) and LDL/HDL ratio (**I**) in the entire RA cohort, 5 mg bid and 10 mg bid treatment arm, respectively, at baseline, and 6 and 12 months of treatment. Asterisks indicate significant changes (*p* < 0.05).

In our cohort of RA patients, TC levels significantly increased after 6 months (5.95 ± 1.15 mmol/L; *p* = 0.003) and 12 months (5.95 ± 1.20 mmol/L; *p* = 0.007) compared with the baseline (5.49 ± 0.92 mmol/L) (Figure 1A). HDL significantly decreased after 6 months (1.62 ± 0.58 mmol/L; *p* = 0.047), but significantly increased after 12 months (1.66 ± 0.51 mmol/L; *p* = 0.004) compared with the baseline (1.64 ± 0.95 mmol/L) (Figure 1B). LDL significantly increased after 6 months (3.67 ± 0.95 mmol/L; *p* = 0.039) and 12 months (3.90 ± 1.12 mmol/L; *p* = 0.003) compared with the baseline (3.43 ± 0.83 mmol/L), and there was also a significant increase between 6 months and 12 months (*p* = 0.035) (Figure 1C). APOA also significantly increased after 6 months (1.76 ± 0.42 g/L; *p* = 0.024) and 12 months (1.79 ± 0.39 g/L; *p* = 0.001) compared with the baseline (1.65 ± 0.42 g/L) (Figure 1D). APOB also showed a significant increase after 6 months (1.19 ± 0.33 g/L; *p* = 0.022) and 12 months (1.21 ± 0.31 g/L; *p* = 0.006) compared with the baseline (1.09 ± 0.25 g/L) (Figure 1E). Lp(a) showed a significant decrease after 6 months (147.53 ± 190.27 mg/L; *p* = 0.013) and 12 months (150.80 ± 202.29 mg/L; *p* = 0.024) compared with the baseline (181.20 ± 237.81 mg/L) (Figure 1F). TG and lipid ratios (TC/HLDL, LDL/HDL) showed no significant changes between baseline and one year upon tofacitinib therapy (Figure 1G, 1H and 1I, respectively). In the treatment group of 5 mg bid tofacitinib, we found no significant changes (data not shown); however, in the treatment group of 10 mg bid tofacitinib we found similar changes (data not shown) to that of the whole cohort (Figure 1).

**Figure 2:**
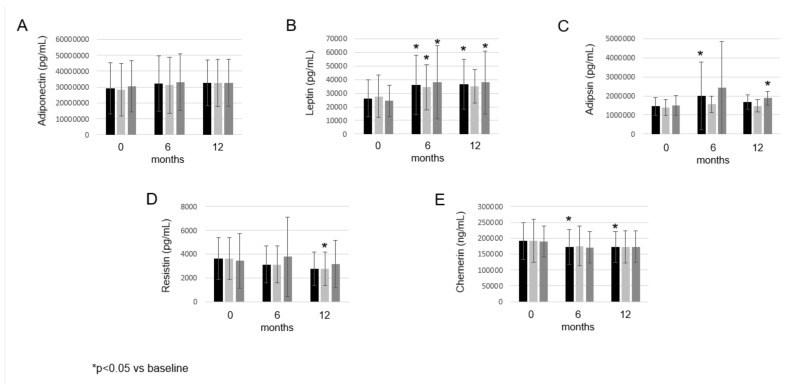
Effects of one-year tofacitinib therapy on adiponectin (**A**), leptin (**B**), adipsin (**C**), resistin (**D**) and chemerin (**E**) levels in the entire RA whole cohort, 5 mg bid and 10 mg bid treatment arm, respectively, at baseline, and 6 and 12 months of treatment. Asterisks indicate significant changes (*p* < 0.05).

Among adipokines, only adiponectin numerically increased after 6 and 12 months compared with the baseline (Figure 2A). Leptin showed a significant increase after 6 months (36,196.97 ± 21,952.19 pg/mL; *p* = 0.001) and 12 months (36,467.00 ± 18,219.75 pg/mL; *p* = 0.003) compared with the baseline (26,071.51 ± 13,592.91 pg/mL) (Figure 2B). Adipsin significantly increased after 6 months (1,995,901.98 ± 1772,069.37 pg/mL; *p* = 0.030), but only numerically increased after 12 months (1,680,141.92 ± 390,567.84 pg/mL; *p* = 0.124) compared with the baseline (1,447,195.50 ± 463,232.82 pg/mL) (Figure 2C). Resistin only numerically decreased after 6 and 12 months compared with the baseline (Figure 2D). Chemerin showed a significant decrease after 6 months (173,193.33 ± 54,900.17 ng/mL; *p* = 0.024) and 12 months (172965.38 ± 48647.12 ng/mL; *p* = 0.040) compared with the baseline (191,196.67 ± 57,747.70 ng/mL) (Figure 2E). In the treatment group of 5 mg bid and 10 mg bid tofacitinib, we found similar changes (data not shown) to that of the whole cohort (Figure 2).

**Figure 3:**
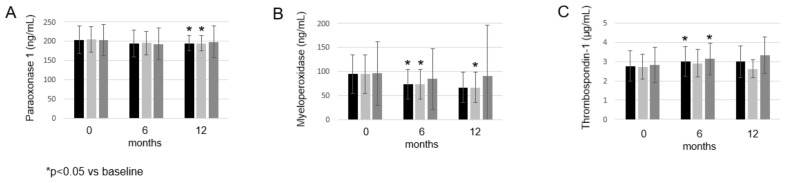
Effects of one-year tofacitinib therapy paraoxonase 1 (**A**), myeloperoxidase (**B**) and thrombospondin-1 (**C**) levels in the entire RA cohort, 5 mg bid and 10 mg bid treatment arm, respectively, at baseline, and 6 and 12 months of treatment. Asterisks indicate significant changes (*p* < 0.05).

PON1 numerically decreased after 6 months (193.79 ± 35.71 ng/mL; *p* = 0.079) and significantly decreased after 12 months (194.10 ± 19.86 ng/mL; *p* = 0.040) compared with the baseline (203.21 ± 36.05 ng/mL) (Figure 3A). MPO significantly decreased after 6 months (73.27 ± 31.54 ng/mL; *p* = 0.028) and numerically after 12 months (66.80 ± 31.84 ng/mL; *p* = 0.058) compared with the baseline (94.46 ± 39.89 ng/mL) (Figure 3B). Finally, TSP-1 significantly increased after 6 months (3.02 ± 0.77 μg/mL; *p* = 0.009), but only numerically after 12 months (2.99 ± 0.82 μg/mL; *p* = 0.182) compared with the baseline (2.77 ± 0.78 μg/mL) (Figure 3C). In the treatment group of 5 mg bid and 10 mg bid tofacitinib, we found similar changes (data not shown) to that of the whole cohort (Figure 3).

### 3.3. Associations of Metabolic Biomarkers with Clinical Parameters, Vascular Pathophysiology and Other Parameters

Several correlations were found between metabolic, clinical and vascular parameters in a simple correlation analysis performed by Pearson’s correlation analysis (data not presented). Correlation analysis was performed for the whole study population, as well as for each treatment arm. Baseline BMI correlated positively with disease activity, CRP, ESR, FMD, PWV, leptin, resistin, PON1 and MPO, while inversely with TC, HDL and APOA (*p* < 0.05). In general, in our study cohort, lipids and lipid ratios variably correlated with other lipids, lipid ratios, adipokines, other metabolic parameters, and clinical and vascular parameters (*p* < 0.05). Not including all the correlations, IMT correlated with age, TC, LDL, adiponectin, chemerin and PWV (*p* < 0.05). FMD correlated with BMI, CRP, TG, TC, Lp(a), HDL, APOA, TC/HDL, LDL/HDL, leptin and adipsin (*p* < 0.05). PWV showed positive correlations with age, BMI, TC, LDL, APOB, LDL/HDL, adipsin, resistin, MPO and IMT, and inverse correlation with HDL (*p* < 0.05). Among adipokines, adiponectin, adipsin, leptin and chemerin correlated with age (*p* < 0.05). Leptin and resistin correlated positively, while adiponectin inversely with CRP (*p* < 0.05). Leptin and resistin correlated with BMI (*p* < 0.05). Adiponectin correlated positively, while chemerin inversely with IMT (*p* < 0.05). Adipsin and resistin correlated with PWV, leptin correlated with FMD, while adipsin also correlated with FMD (*p* < 0.05). Adiponectin, leptin and resistin correlated positively with adipsin (*p* < 0.05). Leptin also correlated with resistin, while adipsin correlated with adiponectin, leptin and resistin (*p* < 0.05). Adiponectin correlated with TSP-1 and PON1, adipsin with TSP-1, PON1 and MPO, resistin with PON1 and MPO, leptin only with PON1, and chemerin only with TSP-1 (*p* < 0.05). Adiponectin also correlated positively with HDL, APOA and APOA/APOB, while inversely with TG, TC/HDL, LDL/HDL and CCP (*p* < 0.05). Leptin correlated inversely with TC, adipsin correlated inversely with RF. Resistin correlated positively with disease activity, ESR, RF and leptin, but inversely with TC and APOA. Chemerin correlated positively with LDL, APOB and LDL/HDL (*p* < 0.05). When examining the correlations with TSP-1, PON1 and MPO, we found that TSP-1 correlated positively with HDL, APOA, adiponectin, adipsin, chemerin and PON1, while inversely with disease activity, TG, TC/HDL, LDL/HDL, RF and ESR (*p* < 0.05). MPO correlated with BMI, CRP, Lp(a), adipsin, resistin, PON1 and PWV (*p* < 0.05). PON1 correlated positively with age, BMI, adiponectin, adipsin, leptin, resistin, TSP-1 and MPO, while inversely with TC/HDL, LDL/HDL, CCP and RF (*p* < 0.05). The correlation analysis found similar associations for the treatment arms as well (data not shown).

In order to determine independent metabolic determinants of FMD, IMT and PWV (Table 2A), as well as independent determinants of our studied metabolic parameters (Table 2B), univariable and multivariable regression analyses were performed for the whole study population, as well as for each treatment arm. In the univariable analysis, FMD positively correlated with BMI, TG, TC, APOB, Lp(a), TC/HDL, LDL/HDL, adipsin and leptin, and inversely with APOA (*p* < 0.05). IMT showed a positive correlation with age, TC and adiponectin, and inversely with chemerin (*p* < 0.05). PWV was positively correlated with age, BMI, adipsin, resistin, MPO and LDL (*p* < 0.05) (Table 2A). The multivariable analysis confirmed the abovementioned associations of FMD with TC/HDL and leptinꓼ IMT with ageꓼ and PWV with age, BMI, resistin and LDL (*p* < 0.05) (Table 2A).

Examining the results of the univariable analysis on determinants of the metabolic markers, we found that BMI correlated with disease activity, CRP, ESR and PWV (*p* < 0.05). TC correlated positively with FMD and PWV, but inversely with BMI, CRP and disease activity (*p* < 0.05). HDL showed inverse correlations with BMI, disease activity, CRP, ESR, CCP, RF, FMD and PWV (*p* < 0.05). LDL was shown to correlate only with PWV, while TG with FMD (*p* < 0.05). APOA was correlated inversely with BMI, disease activity, ESR, CRP, RF and FMD (*p* < 0.05), while APOB correlated positively with disease activity, ESR, RF, FMD and PWV (*p* < 0.05). Lp(a)correlated only with age and FMD (*p* < 0.05). Among lipid ratios, TC/HDL showed a correlation with disease activity, CRP, ESR, RF and FMD (*p* < 0.05). LDL/HDL correlated with disease activity, ESR, RF and PWV (*p* < 0.05). APOA/APOB inversely correlated with disease activity, ESR and RF (*p* < 0.05). Among adipokines, adiponectin correlated positively with age and IMT, but inversely with CRP, CCP and RF (*p* < 0.05). Leptin correlated positively with age, BMI, CRP and FMD (*p* < 0.05). Adipsin was shown to correlate positively with age, PWV and FMD, while inversely with RF (*p* < 0.05). Resistin correlated with BMI, CRP, ESR and PWV (*p* < 0.05). Chemerin showed an inverse correlation with age and IMT (*p* < 0.05). Among other metabolic markers, MPO correlated positively with BMI, CRP, disease duration and PWV (*p* < 0.05), PON1 correlated inversely with CRP and RF, but positively with age and BMI (*p* < 0.05), and TSP-1 correlated inversely with disease activity, RF and ESR (*p* < 0.05). Finally, the calculated leptin/adiponectin ratio correlated positively with CCP and FMD, but inversely with age, IMT and PWV (*p* < 0.05) (Table 2B). A number of these associations were also confirmed by multivariable analysis. These included associations of BMI with CRP and PWVꓼ inverse association of TC with CRPꓼ inverse association of HDL with ESR, FMD and PWVꓼ inverse association of APOA with ESR, CRP and RFꓼ APOB with FMD and PWVꓼ Lp(a) with age and FMDꓼ TC/HLD with RFꓼ LDL/HDL with disease activity, RF and PWVꓼ inverse association of APOA/APOB with ESRꓼ adiponectin with ageꓼ inverse association of adiponectin with CCPꓼ leptin with age, BMI and FMDꓼ adipsin with PWVꓼ resistin with CRPꓼ inverse association of chemerin with IMTꓼ MPO with disease duration ad PWVꓼ PON1 with BMI; and inverse association of PON1 with age and RFꓼ inverse association of TSP-1 with RFꓼ leptin/adiponectin ratio with FMD; and inverse association of leptin/adiponectin ratio with age and IMT (*p* < 0.05) (Table 2B). Similar associations were found in the treatment arms (data not shown).

Finally, RM-ANOVA analysis was performed to identify combined determinants of changes in metabolic marker levels between baseline and 12 months (Table 3.) Adiponectin changes overtime were associated with treatment and PWV (*p* = 0.023). Leptin changes correlated with treatment and age (*p* = 0.043), as well as treatment and CRP (*p* = 0.005). Resistin changes were associated with treatment and BMI (*p* = 0.005). Changes in TSP-1 correlated with treatment and CRP (*p* = 0.029). PON1 changes were associated with treatment and CRP (*p* = 0.032), as well as treatment and ESR (*p* = 0.022). Finally, MPO changes correlated with treatment and disease duration (*p* = 0.038), as well as with treatment and CCP (*p* = 0.046) (Table 3).

## 4. Discussion

To our best knowledge, this may be the first one-year, prospective study assessing lipids, adipokines and other metabolic parameters, including in relation to clinical parameters and vascular function, in RA patients undergoing tofacitinib therapy. We also compared 5 mg bid and 10 mg bid dosing.

In the very same patient cohort, as reported previously, one-year tofacitinib therapy was shown to be effective, as it resulted in significantly decreased disease activity as well as CRP and improved HAQ [51,53,59]. Regarding effects on vascular pathophysiology, we also refer to our previous report. Briefly, FMD and PWV showed no changes, while IMT showed an increase overtime [53].

### 4.1. Effects of Tofacitinib Therapy on Circulating Metabolic Biomarkers

In the present study, one-year tofacitinib treatment increased lipids and lipoproteins, including TC, HDL, LDL, APOA and APOB, but decreased the pro-atherogenic Lp(a). TG and lipid ratios did not change overtime. Similar to our findings, tofacitinib therapy in combination with DMARDs or as a monotherapy was previously reported to increase LDL and HDL levels [5,15,16]. In our study, lipid ratios (TC/HDL and LDL/HDL) showed no significant changes indicating that lipid elevations observed upon tofacitinib therapy may not have important clinical relevance for CV disease. Only very slight change in the LDL/HDL ratio was previously reported [15]. In our treatment group of 5 mg bid tofacitinib, no significant changes were found; however, in the treatment group of 10 mg bid tofacitinib, similar changes to that of the whole cohort have been observed. We may speculate that it is due to the smaller mean BMI in the group of 5 mg bid (28.82 ± 5.63) compared with that of the 10 mg bid (31.03 ± 8.01). Among adipokines, tofacitinib treatment significantly increased leptin and adipsin levels, while it decreased chemerin levels. Adiponectin and resistin showed no significant changes overtime. PON1 and MPO levels significantly decreased, while TSP-1 significantly increased overtime. The suppression of chemerin and MPO levels indicate the effects of tofacitinib on inflammation. The increased production of the angiostatic TSP-1 suggests the favorable effect of JAK inhibition on inflammation-associated neovascularization. We have not found any other studies on tofacitinib effects on adipokines, MPO or TSP-1. In one study carried out in a patient population different from ours, tofacitinib therapy increased PON1 levels in patients with active RA [59]. In our study, the decreased PON1 concentration after 6 months is an unexpected result, since in general, dampening of inflammation increases PON1 expression. Although the safety of tofacitinib is well documented, JAK inhibitors have been associated with a low rate of serum liver enzyme elevations during therapy; however, this has not been linked to cases of clinically apparent acute liver injury. A former study found significantly lower PON1 levels in patients with acute liver disease as compared with controls [60]. Although the decrease in PON1 level was statistically significant during the 6-month follow-up, the changes might not be clinically relevant, and may be explained by the moderate and harmless liver involvement. Further studies on larger patient populations are needed to clarify the long-term effect of tofacitinib on PON1 expression.

### 4.2. Associations of Metabolic Biomarkers with Clinical Parameters, Vascular Pathophysiology and Other Parameters

In the correlation analysis, BMI correlated positively with disease activity, CRP, ESR, FMD, PWV, leptin, resistin, PON1 and MPO, while inversely with TC, HDL and APOA. Univariable regression analysis found a possible association of BMI with disease activity, CRP, ESR and PWV, while multivariable analysis confirmed association with CRP and PWV.

Lipids and lipid ratios variably correlated with other lipids, lipid ratios, adipokines, and other metabolic parameters, as well as clinical and vascular parameters. Univariable regression analysis found various possible associations of lipids and lipid ratios, while multivariable analysis confirmed inverse associations of TC with CRP; HDL with ESR, FMD and PWV; APOA with ESR, CRP and RF; and APOA/APOB with ESRꓼ and positive associations of APOB with FMD and PWV; Lp(a) with age and FMD; TC/HLD with RF; and LDL/HDL with disease activity, RF and PWV.

Among adipokines, adiponectin correlated positively with age, HDL, APOA, APOA/APOB, IMT, adipsin, TSP-1 and PON1, while inversely with CRP, TG, TC/HDL, LDL/HDL and CCP. Univariable regression analysis showed a possible positive association with age and IMT, but an inverse association with CRP, CCP and RF. Multivariable analysis confirmed a positive association with age and inverse association with CCP. Adiponectin was previously positively associated with age and disease activity, while inversely associated with BMI [26,61] in rheumatic conditions. In our study, adiponectin changes overtime were associated with treatment and PWV indicating the relationship of adiponectin and vascular stiffness.

Leptin correlated positively with age, CRP, BMI, FMD, adipsin, resistin and PON1, while inversely with TC. Univariable regression analysis showed possible positive association with age, BMI, CRP and FMD. Multivariable analysis confirmed association with age, BMI and FMD. Leptin was previously correlated with BMI, disease duration, disease activity, ESR and CRP [26,62,63]. In patients with RA undergoing anti-TNF therapy, leptin serum levels showed a positive correlation with BMI and VCAM-1; however, no significant correlations were observed between leptin levels and age, disease duration, ESR, CRP, DAS28, lipids, insulin sensitivity, resistin, adiponectin, ghrelin or the cumulative prednisone dose at the time of the study. (Ref. Anti-TNF-alpha therapy does not modulate leptin in patients with severe rheumatoid arthritis. Clin Exp Rheumatol. 2009 Mar-Apr;27(2):222-8. PMID: 19473561). In our study, leptin changes correlated with treatment and age, as well as treatment and CRP.

Adipsin showed positive correlation with age, PWV, FMD, adiponectin, leptin, resistin, TSP-1, PON1 and MPO, but an inverse correlation with RF. Univariable regression analysis showed a possible positive association with age, PWV and FMD, and inverse association with RF. Multivariable analysis confirmed association with PWV. Adipsin was previously reported to correlate with BMI and disease activity [64].

Resistin correlated positively with BMI, disease activity, CRP, ESR, RF, PWV, adipsin, leptin, PON1 and MPO, while inversely with TC and APOA. Univariable regression analysis showed a possible positive association with BMI, CRP, ESR and PWV. Multivariable analysis confirmed association with CRP. Resistin was previously associated with CRP and disease activity [23,65,66]. In our study, resistin changes were associated with treatment and BMI.

Chemerin showed positive correlation with age, LDL, APOB, LDL/HDL and TSP-1, while inverse correlation with IMT. Univariable regression analysis showed a possible inverse correlation with age and IMT. Multivariable analysis confirmed association with IMT. Chemerin was previously correlated with disease activity, BMI, CRP, RF, ESR and CCP [67,68].

MPO showed a positive correlation with BMI, CRP, Lp(a), adipsin, resistin, PON1 and PWV. Univariable regression analysis showed a possible positive association with BMI, CRP, disease duration and PWV. Multivariable analysis confirmed association with disease duration and PWV. Similar to our findings, a paper reported on positive correlation with CRP, but also with disease activity [69]. In our study, MPO changes correlated with treatment and disease duration, as well as with treatment and CCP.

TSP-1 correlated positively with HDL, APOA, adiponectin, adipsin, chemerin and PON1, while inversely with disease activity, TG, TC/HDL, LDL/HDL, RF and ESR. Univariable regression analysis showed a possible inverse association with disease activity, RF and ESR. Multivariable analysis confirmed inverse association with RF. TSP-1 was previously associated with disease activity and ESR [70]. Changes in TSP-1 correlated with treatment and CRP.

Tofacitinib therapy was previously reported to increase PON1 levels [59]. In our study, PON1 correlated positively with age, BMI, adiponectin, adipsin, leptin, resistin, TSP-1 and MPO, while inversely with TC/HDL, LDL/HDL, CCP and RF. Univariable regression analysis showed possible positive association with age and BMI, while inverse association with CRP and RF. Multivariable analysis confirmed positive association with BMI, while inverse association with age and RF. Previous studies reported an inverse association with RF, CCP and disease activity [59,71]. Finally, PON1 changes were associated with treatment and CRP, as well as with treatment and ESR.

This study has certain advantages and disadvantages. It is a complex follow-up study assessing a number of metabolic parameters, together with previous findings of clinical efficacy and vascular pathophysiology in the same cohort. It also compares two therapeutic dosing modalities. As a limitation, a relatively small sample size was used; however, a larger number of tests and measurements would have been a lot harder to process, and more time- and resource-consuming to carry out on a bigger sample. Patients with a potentially positive history of CV disease and diabetes mellitus were also included; however, these patients had no complaints of this nature, as their disease was controlled.

## 5. Conclusions

To conclude, one-year tofacitinib therapy significantly increased TC, HDL, LDL, APOA, APOB, leptin, adipsin and TSP-1, while it significantly decreased Lp(a), chemerin, PON1 and MPO levels. TG, lipid indices (TC/HDL and LDL/HDL), adiponectin and resistin showed no significant changes. We found numerous significant associations between lipids, clinical parameters, other metabolic markers (PON1, MPO, TSP-1 and adipokines) and vascular pathophysiology. To our knowledge, this may be the first study assessing lipids and other metabolic markers together with clinical parameters as well as markers of vascular pathophysiology. This kind of complex assessment may extend our knowledge about these metabolic parameters as well as be utilized in clinical practice to determine and monitor the CV status of patients in relation with clinical response.

## Figures and Tables

**Table 1:** Patient characteristics.

	Tofacitinib 5 mg Bid	Tofacitinib 10 mg Bid	Total
*n*	15	15	30
female:male	14:1	13:2	27:3
age (mean ± SD) (range), years	52.3 ± 11.4 (27–69)	53.3 ± 8.8 (34–69)	52.8 ± 10.0 (27–69)
disease duration (mean ± SD) (range), years	6.3 ± 4.7 (1–15)	7.1 ± 4.9 (2–21)	7.7 ± 5.0 (1–21)
smoking (current)	4	3	7
positive history of cardiovascular disease	3	3	6
body mass index (BMI; mean ± SD), kg/m^2^	28.82 ± 5.63	31.03 ± 8.01	29.93 ± 6.90
obesity (BMI > 30 kg/m^2^)	4	6	10
diabetes mellitus history	1	1	2
hypertension history	5	10	15
rheumatoid factor positivity, *n* (%)	12 (80)	12 (80)	24 (80)
ACPA positivity, *n* (%)	13 (87)	11 (73)	24 (80)
DAS28 (baseline) (mean ± SD)	4.80 ± 0.69	5.29 ± 0.79	5.05 ± 0.77
MTX	9	7	16
Sulfasalazine	0	1	1
Leflunomide	2	2	4
MTX + sulfasalazin	1	0	1
Leflunomide + sulfasalazin	0	1	1

Abbreviations: ACPA, anti-citrullinated protein antibodies; BMI, body mass index; DAS28, 28-joint disease activity score; MTX, methotrerate; SD, standard deviation.

**Table 2:** Univariable and Multivariable Regression Analysis of Metabolic and Vascular Associations.

(A). Determination of Vascular Pathophysiology by Metabolic Markers									
Dependent Variable	Independent Variable	Univariable Analysis				Multivariable Analysis			
		β	*p*	B	CI 95%	β	*p*	B	CI 95%
FMD-0	Lp(a)-0	0.435	0.016	0.007	0.001–0.013				
FMD-6	BMI	0.411	0.024	0.238	0.034–0.442				
	APOA-0	−0.411	0.024	−3.947	−7.338–−0.555				
	adipsin-6	0.466	0.009	0					
	leptin-0	0.412	0.024	0					
	leptin-6	0.670	<0.001	0		0.670	<0.001	0	
FMD-12	TG-12	0.375	0.041	1.077	0.045–2.109				
	TC-6	0.383	0.037	1.027	0.067–1.988				
	TC-12	0.368	0.045	0.952	0.021–1.883				
	APOB-12	0.367	0.046	3.646	0.073–7.218				
	TC/HDL-0	0.433	0.017	0.891	0.174–1.809	0.433	0.017	0.891	0.174–1.809
	TC/HDL-6	0.398	0.029	0.476	0.051–0.900				
	TC/HDL-12	0.376	0.041	0.951	0.043–1.859				
	LDL/HDL-0	0.373	0.042	1.016	0.038–1.993				
IMT-0	age	0.711	<0.001	0.008	0.005–0.011	0.711	<0.001	0.008	0.005–0.011
	adiponectin-0	0.524	0.003	0					
	chemerin-0	−0.368	0.025	0					
IMT-6	age	0.649	<0.001	0.007	0.004–0.011	0.649	<0.001	0.007	0.004–0.011
	TC-0	0.410	0.025	0.051	0.007–0.095				
	adiponectin-0	0.504	0.005	0					
IMT-12	age	0.419	0.021	0.007	0.001–0.013				
PWV-0	age	0.362	0.049	0.060	0–0.120	0.330	0.027	0.055	0.007–0.103
	BMI	0.513	0.004	0.123	0.043–0.203	0.342	0.030	0.082	0.009–0.156
	adipsin-0	0.418	0.022	0					
	resistin-0	0.501	0.005	0		0.400	0.012	0	
	MPO-0	0.373	0.043	0.011	0–0.023				
PWV-6	age	0.501	0.005	0.081	0.027–0.136	0.436	0.008	0.071	0.020–0.122
	LDL-6	0.452	0.012	0.775	0.183–2.368	0.377	0.020	0.647	0.109–1.185
PWV-12	age	0.543	0.002	0.091	0.036–0.145				
**(B). Determination of Metabolic Markers by Clinical and Other Parameters**									
**Dependent** **Variable**	**Independent Variable**	**Univariable Analysis**				**Multivariable Analysis**			
		**β**	**p**	**B**	**CI 95%**	**β**	***p***	**B**	**CI 95%**
BMI	DAS28-0	0.384	0.036	3.444	0.241–6.647				
	CRP-0	0.383	0.037	0.178	0.012–0.344	0.326	0.044	0.151	0.005–0.298
	ESR-0	0.371	0.044	0.117	0.003–0.231				
	PWV-0	0.513	0.004	2.132	0.750–3.514	0.474	0.005	1.970	0.655–3.285
TC-6	BMI	−0.367	0.046	−0.061	−0.122–−0.001				
	CRP-6	−0.414	0.023	−0.090	−0.167–−0.013	−0.414	0.023	−0.090	−0.167–−0.013
TC-12	BMI	−0.365	0.047	−0.063	−0.126–−0.001				
	CRP-6	−0.398	0.029	−0.090	−0.171–−0.010	−0.398	0.029	−0.090	−0.171–−0.010
	FMD-12	0.368	0.045	0.142	0.003–0.282				
	PWV-6	0.375	0.041	0.276	0.012–0.541				
HDL-6	BMI	−0.485	0.007	−0.041	−0.069–−0.012				
	DAS28-0	−2.640	0.013	−0.337	−0.599–−0.076				
	CRP-0	−0.419	0.021	−0.016	−0.030–−0.003				
	CRP-6	−0.398	0.029	−0.044	−0.083–−0.005				
	ESR-0	−0.518	0.003	−0.014	−0.023–−0.005	−0.420	0.007	−0.011	−0.019–−0.003
	RF-0	−0.449	0.013	−0.001	−0.002–0				
	FMD-0	−0.379	0.039	−0.057	−0.110–−0.003	−0.416	0.006	−0.062	−0.104–−0.020
	PWV-0	−0.408	0.025	−0.143	−0.267–−0.019	−0.329	0.031	−0.115	−0.220–−0.011
HDL-12	BMI	−0.478	0.008	−0.035	−0.061–−0.010				
	DAS28-0	−0.418	0.022	−0.277	−0.510–−0.044				
	CRP-0	−0.487	0.006	−0.017	−0.028–−0.005				
	CRP-6	−0.413	0.023	−0.400	−0.074–−0.006				
	CRP-12	−0.392	0.032	−0.026	−0.050–−0.002				
	ESR-0	−0.538	0.002	−0.013	−0.020–−0.005	−0.538	0.002	−0.013	−0.020–−0.005
	CCP-0	−0.370	0.044	0	0				
	RF-0	−0.509	0.004	−0.001	−0.002–0				
	RF-6	−0.439	0.015	−0.001	−0.002–0				
	RF-12	−0.439	0.015	−0.001	−0.002–0				
	FDM-6	−0.401	0.028	−0.051	−0.097–−0.006				
	PWV-0	−0.368	0.046	−0.113	−0.224–−0.002				
LDL-6	PWV-6	0.452	0.012	0.263	0.062–0.465				
LDL-12	PWV-6	0.496	0.005	0.343	0.111–0.576				
TG-12	FMD-12	0.375	0.041	0.130	0.005–0.255				
APOA-0	BMI	−0.381	0.038	−0.023	−0.045–−0.001				
	DAS28-0	−0.370	0.044	−0.200	−0.394–−0.006				
	ESR-0	−0.488	0.006	−0.009	−0.016–−0.003	−0.488	0.006	−0.009	−0.016–−0.003
	CRP-0	−0.442	0.015	−0.012	−0.022–0.003				
	RF-0	−0.452	0.012	−0.001	−0.002–0				
APOA-6	BMI	−0.449	0.013	0.028	−0.049–−0.006				
	DAS28-0	−0.374	0.041	−0.206	−0.404–−0.009				
	CRP-6	−0.425	0.019	−0.034	−0.062–−0.006				
	ESR-0	−0.506	0.004	−0.010	−0.016–−0.003	−0.506	0.004	−0.010	−0.016–−0.003
	RF-0	−0.391	0.033	−0.001	−0.001–0				
APOA-12	BMI	−0.451	0.012	−0.025	−0.045–−0.006				
	CRP-0	−0.433	0.017	−0.011	−0.020–−0.002				
	CRP-6	−0.469	0.009	−0.034	−0.059–−0.009	−0.497	0.003	−0.036	−0.059–−0.014
	CRP-12	−0.434	0.017	−0.022	−0.039–−0.004				
	ESR-0	−0.433	0.017	−0.008	−0.014–−0.002				
	ESR-12	−0.389	0.033	−0.007	−0.040–−0.001				
	RF-0	−0.387	0.035	−0.001	−0.001–0	−0.420	0.009	−0.001	−0.001–0
	RF-6	−0.379	0.039	−0.001	−0.002–0				
	RF-12	−0.369	0.045	−0.001	−0.002–0				
	FMD-6	−0.366	0.046	−0.036	−0.070–0.001				
APOB-6	RF-0	0.421	0.021	0.001	0–0.001				
APOB-12	DAS28-0	0.380	0.038	0.154	0.009–0.300				
	ESR-6	0.384	0.036	0.006	0–0.011				
	FMD-12	0.367	0.046	0.037	0.001–0.073	0.557	0.001	0.056	0.027–0.086
	PWV-6	0.463	0.010	0.089	0.023–0.155	0.631	<0.001	0.121	0.065–0.178
TC/HDL-0	DAS28-0	0.484	0.007	0.947	0.283–1.610				
	CRP-0	0.233	0.215	0.024	−0.015–0.062				
	ESR-0	0.468	0.009	0.032	0.009–0.056				
	RF-0	0.575	0.001	0.004	0.002–0.006	0.575	0.001	0.004	0.002–0.006
TC/HDL-6	RF-0	0.385	0.036	0.005	0–0.009				
TC/HDL-12	DAS28-0	0.463	0.010	0.737	0.191–1.283				
	ESR-0	0.502	0.005	0.028	0.009–0.047				
	RF-0	0.583	0.001	0.003	0.002–0.005	0.583	0.001	0.003	0.002–0.005
	RF-6	0.394	0.031	0.003	0–0.005				
	RF-12	0.410	0.025	0.003	0–0.005				
	FMD-12	0.376	0.041	0.148	0.007–0.290				
LDL/HDL-0	DAS28-0	0.515	0.004	0.762	0.271–1.253	0.356	0.034	0.527	0.042–1.013
	ESR-0	0.472	0.008	0.025	0.007–0.043				
	RF-0	0.551	0.002	0.003	0.001–0.005	0.414	0.015	0.002	0–0.004
LDL/HDL-6	DAS28-0	0.424	0.020	0.653	0.112–1.194				
	ESR-0	0.400	0.028	0.022	0.002–0.041				
	RF-0	0.528	0.003	0.003	0.001–0.005	0.528	0.003	0.003	0.001–0.005
LDL/HDL-12	DAS28-0	0.487	0.006	0.638	0.196–1.081				
	ESR-0	0.496	0.005	0.023	0.007–0.039				
	RF-0	0.567	0.001	0.003	0.001–0.004	0.518	0.002	0.002	0.001–0.004
	RF-6	0.414	0.023	0.002	0–0.004				
	RF-12	0.447	0.013	0.003	0.001–0.005				
	PWV-6	0.404	0.027	0.251	0.031–0.470	0.326	0.036	0.202	0.015–0.390
APOA/APOB-0	DAS28-0	−0.472	0.009	−0.400	−0.689–−0.011				
	ESR-0	−0.481	0.007	−0.014	−0.025–−0.004	−0.481	0.007	−0.014	−0.025–−0.004
	RF-0	−0.436	0.016	−0.001	−0.002–0				
APOA/APOB-6	DAS28-0	−0.433	0.017	−0.386	−0.697–−0.075				
	ESR-0	−0.475	0.008	−0.015	−0.026–−0.004	−0.475	0.008	−0.015	−0.026–−0.004
	RF-0	−0.460	0.011	−0.001	−0.003–0				
APOA/APOB-12	DAS28-0	−0.540	0.002	−0.387	−0.620–−0.153				
	ESR-0	−0.563	0.001	−0.014	−0.022–−0.006	−0.563	0.001	−0.014	−0.022–−0.006
	ESR-6	−0.442	0.014	−0.012	−0.021–−0.003				
	ESR-12	−0.460	0.011	−0.012	−0.021–−0.003				
	RF-0	−0.460	0.011	−0.001	−0.002–0				
	RF-12	−0.373	0.042	−0.001	−0.002–0				
Lp(a)-0	age	0.363	0.048	8.625	0.063–17.187	0.368	0.028	8.746	1.045–16.448
	FMD-0	0.435	0.016	26.543	5.287–47.799	0.439	0.010	26.804	7.018–46.889
Lp(a)-6	age	0.392	0.032	7.440	0.675–14.206	0.397	0.018	0.753	1.402–13.665
	FMD-0	0.418	0.022	20.385	0.322–37.547	0.422	0.012	20.609	4.857–36.361
Lp(a)-12	age	0.384	0.036	7.752	0.534–14.970	0.388	0.023	7.844	1.180–14.508
	FMD-0	0.388	0.034	20.149	1.643–38.654	0.393	0.021	20.382	13.263–37.502
adiponectin-0	age	0.842	0.002	874,351.2	348,950.2–139,752.1	0.842	0.002	874,351.2	348,950.2–139,752.1
	CRP-0	−0.379	0.039	−412,798.6	−802,946.1–−22,651.2				
	IMT-0	0.524	0.003	76,466,231.7	28,396,912.8–124,535,550.6				
adiponectin-6	age	0.438	0.015	761,832.4	157,051.5–1,366,613.3	0.438	0.015	761,832.4	157,051.5–1,366,613.3
	IMT-0	0.370	0.044	58,042,849.8	1,572,670.8–114,513,028.9				
adiponectin-12	age	0.403	0.041	664,577.1	28,099.4–1,301,054.8				
	CCP-0	−0.439	0.025	−5542.26	−10,318.37–−766.15	−0.439	0.025	−5542.26	−10,318.37–−766.15
	CCP-6	−0.412	0.041	−4837.45	−9451.85–−223.06				
	CCP-12	−0.434	0.027	−5230.24	−9801.93–−658.55				
	RF-12	−0.406	0.040	−33,318.39	−64,947.41–−1689.37				
leptin-0	age	0.418	0.022	566.75	89.48–1044.01	0.330	0.007	448.34	130.21–766.47
	BMI-0	0.739	<0.001	1455.82	941.45–1970.19	0.697	<0.001	1374.32	912.22–1836.42
leptin-6	BMI-0	0.694	<0.001	2210.08	1323.26–3096.89	0.694	<0.001	2210.08	1323.26–3096.89
	CRP-6	0.487	0.006	2019.18	617.30–3421.06				
	FMD-0	0.369	0.045	2078.29	52.89–4103.69				
	FMD-6	0.670	<0.001	3685.28	2105.90–5264.65				
leptin-12	BMI	0.622	0.001	1592.52	748.66–2436.38	0.411	0.007	1052.07	311.99–1792.15
	CRP-6	0.592	0.001	1945.24	830.83–3059.66				
	CRP-12	0.621	0.001	1375.82	644.21–2107.44				
	FMD-6	0.693	<0.001	3002.41	1686.23–4318.61	10.529	0.001	2290.75	1037.58–3543.91
adipsin-0	age	0.366	0.047	16,935.7	278.5–33,592.9				
	PWV-0	0.418	0.022	116,643.3	18,420.5–214,866.1	0.418	0.022	116643.3	18420.5–214,866.1
adipsin-6	FMD-6	0.466	0.009	206,893.2	54,898.5–358,887.9				
adipsin-12	RF-6	−0.445	0.023	−958.74	−1771.75–−145.73				
	RF-12	−0.406	0.040	−904.56	−1761.96–−47.15				
resistin-6	BMI	0.590	0.001	220.179	103.653–336.706				
	CRP-6	0.724	<0.001	351.619	221.862–481.375	0.724	<0.001	351.619	221.862–481.375
	PWV-0	0.431	0.017	668.222	126.601–1209.842				
resistin-12	BMI	0.479	0.013	114.277	25.951–202.603				
	CRP-0	0.458	0.019	49.007	8.919–89.096				
	CRP-6	0.687	<0.001	210.528	116.751–304.306				
	CRP-12	0.704	<0.001	145.517	83.652–207.382	0.704	<0.001	145.517	83.652–207.382
	ESR-0	0.417	0.034	33.273	2.705–63.841				
chemerin-0	IMT-0	−0.368	0.045	−191,843.4	−379,232.6–−4454.2				
chemerin-12	age	−0.457	0.019	−2539.24	−4623.04–−455.44				
	IMT-0	−0.476	0.014	−209,528.3	−372,585.3–−46,471.3	−0.476	0.014	−209,528.3	−372,585.3–−46,471.3
MPO-0	PWV-0	0.373	0.043	12.089	0.436–23.731				
MPO-6	BMI	0.410	0.024	2.937	0.407–5.466				
	CRP-6	0.412	0.024	3.847	0.557–7.136				
	PWV-0	0.536	0.002	15.972	6.245–25.699	0.536	0.002	15.972	6.245–25.699
MPO-12	Dis. duration	0.469	0.016	6.914	1.426–12.402	0.415	0.024	6.119	0.898–11.339
	PWV-0	0.417	0.034	18.604	1.533–35.677	0.354	0.050	15.785	0.003–31.568
PON1-0	CRP-0	−0.393	0.032	−0.954	−1.818–−0.090				
	RF-0	−0.518	0.003	−0.086	−0.141–−0.031	−0.518	0.003	−0.086	−0.141–−0.031
PON1-6	age	0.370	0.044	1.317	0.035–2.600	−0.563	0.001	−0.093	−0.141–−0.044
	RF-0	−0.520	0.003	−0.085	−0.140–−0.031	−0.563	0.001	−0.093	−0.141–−0.044
	RF-6	−0.424	0.020	−0.086	−0.157–−0.015				
PON1-12	BMI-0	0.603	0.001	2.667	1.182–4.152	0.562	<0.001	2.485	1.257–3.713
	CRP-6	0.478	0.014	2.711	0.612–4.810				
	RF-0	−0.524	0.006	−0.075	−0.127–−0.024	−0.476	0.002	−0.068	−0.108–−0.038
	RF-6	−0.437	0.026	−0.076	−0.141–−0.010				
	RF-12	−0.459	0.018	−0.082	−0.149–−0.015				
TSP-1-0	DAS28-0	-0.419	0.021	−0.425	−0.781–−0.068				
	RF-0	−0.426	0.019	−0.002	−0.003–0	−0.426	0.019	−0.002	−0.003–0
TSP-1-6	ESR-0	−0.369	0.045	−0.013	−0.026–0				
	RF-0	−0.407	0.025	−0.001	−0.003–0	−0.407	0.025	−0.001	−0.003–0
TSP-1-12	ESR-0	−0.504	0.009	−0.019	−0.039–−0.005				
	RF-0	−0.535	0.005	−0.002	−0.003–−0.001				
	RF-6	−0.554	0.003	−0.002	−0.004–−0.001				
	RF-12	−0.591	0.001	−0.003	−0.004–−0.001	−0.591	0.001	−0.003	−0.004–−0.001
leptin/adiponectin-0	IMT-0	−0.426	0.019	−0.005	−0.009–−0.001				
leptin/adiponectin-6	FMD-6	0.490	0.006	0					
	IMT-0	−0.472	0.008	−0.006	−0.010–−0.002	−0.472	0.008	−0.006	−0.010–−0.002
	IMT-6	−0.412	0.023	−0.005	−0.009–−0.001				
leptin/adiponectin-12	age	−0.558	0.003	0		−0.520	0.001	0	
	CCP-0	0.415	0.035	0					
	CCP-6	0.421	0.036	0					
	CCP-12	0.415	0.035	0					
	FMD-6	0.567	0.003	0		0.529	0.001	0	
	IMT-0	−0.461	0.018	−0.006	−0.012–−0.001				
	IMT-6	−0.449	0.021	−0.006	−0.011–−0.001				
	PWV-6	−0.396	0.045	0	−0.001–0				

Only significant correlations are listed in this table. Abbreviations: APOA, apolipoprotein Aꓼ APOB, apolipoprotein Bꓼ BMI, body mass indexꓼ CCP, citrullinated proteinꓼ CRP, C-reactive proteinꓼ DAS, disease activityꓼ ESR, erythrocyte sedimentation rate; FMD, flow-mediated vasodilationꓼ HDL, high-density lipoproteinꓼ IMT, intima-media thicknessꓼ LDL, low-density lipoproteinꓼ Lp(a), lipoprotein (a)ꓼ MPO, myeloperoxidaseꓼ PON1, paraoxonase 1ꓼ PWV, pulse-wave velocityꓼ RF, rheumatoid factorꓼ TC, total cholesterolꓼ TG, triglycerideꓼ TSP-1, thrombospondin-1ꓼ the numbers −0, −6 and −12 indicate values at baseline and after 6 and 12 months of treatment.

**Table 3:** Significant results of the general linear model repeated measures analysis of variance (RM-ANOVA) test determining the effects of treatment and other independent variables on metabolic parameters as dependent variables.

Dependent Variable	Effect	F	*p*	Partial η^2^
Adiponectin 0-6-12	Treatment × PWV	4.482	0.023	0.280
Leptin 0-6-12	Treatment × age Treatment × CRP	3.617 6.682	0.043 0.005	0.239 0.368
Resistin 0-6-12	Treatment × BMI	6.587	0.005	0.364
Thrombospondin 0-6-12	Treatment × CRP	4.147	0.029	0.265
PON1 0-6-12	Treatment × CRP Treatment × ESR	4.125 4.525	0.032 0.022	0.147 0.282
MPO 0-6-12	Treatment × disease duration Treatment × CCP	3.507 3.597	0.038 0.046	0.128 0.130

Only significant results are listed in this table. Abbreviations: BMI, body mass indexꓼ CCP, citrullinated proteinꓼ CRP, C-reactive proteinꓼ ESR, erythrocyte sedimentation rateꓼ MPO, myeloperoxidaseꓼ PON1, paraoxonase1ꓼ PWV, pulse wave velocity. The numbers 0-6-12 indicate values at baseline and after 6 and 12 months of treatment.

## Data Availability

Data are available from the authors upon request.
